# Listen and learn

**DOI:** 10.7554/eLife.88989

**Published:** 2023-06-07

**Authors:** Sarah E London

**Affiliations:** 1 Department of Psychology, Institute for Mind and Biology and Neuroscience Institute, University of Chicago Chicago United States

**Keywords:** sensorimotor plasticity, songbird, gene expression, single cell sequencing, learned behavior, Other

## Abstract

In songbirds, deafening leads to changes in gene expression which have now been mapped at the single-cell level across the neural circuit involved in song production.

**Related research article** Colquitt BM, Li K, Green F, Veline RJ, Brainard MS. 2023. Neural circuit-wide analysis of gene expression during deafening-induced destabilization of birdsong. *eLife* **12**:e85970. doi: 10.7554/eLife.85970.

Imagine trying to master the violin with an instrument that made no sound, or trying to play football with a foot that was numb. Both would seem almost impossible because learning motor skills depends heavily on sensory feedback. As well as supporting learning, sensory information is also required to maintain these acquired motor behaviors. For instance, people who become deaf later in life gradually lose the ability to speak clearly, even though they have done so for years (Lane and Webster, 1991).

The same is true for songbirds (Marler and Doupe, 2000). The highly structured songs of these birds are learned vocalizations that become disrupted if an adult songbird loses the ability to hear (Nordeen and Nordeen, 1992; Okanoya and Yamaguchi, 1997; Woolley and Rubel, 1997). Despite inextricable links between sensory input and motoric output, it is still unclear how sensory loss affects brain circuits and, as a consequence, how it affects any learned behaviors.

Studying the impact of sensory loss can be a challenge, as learned behaviors often involve several regions of the brain. Moreover, different brain regions can harbor diverse sets of cell types that must work together. To complicate matters, these cell types are distributed differently across various brain areas, and the set of genes expressed by each cell type also varies (Figure 1).

**Figure 1.**
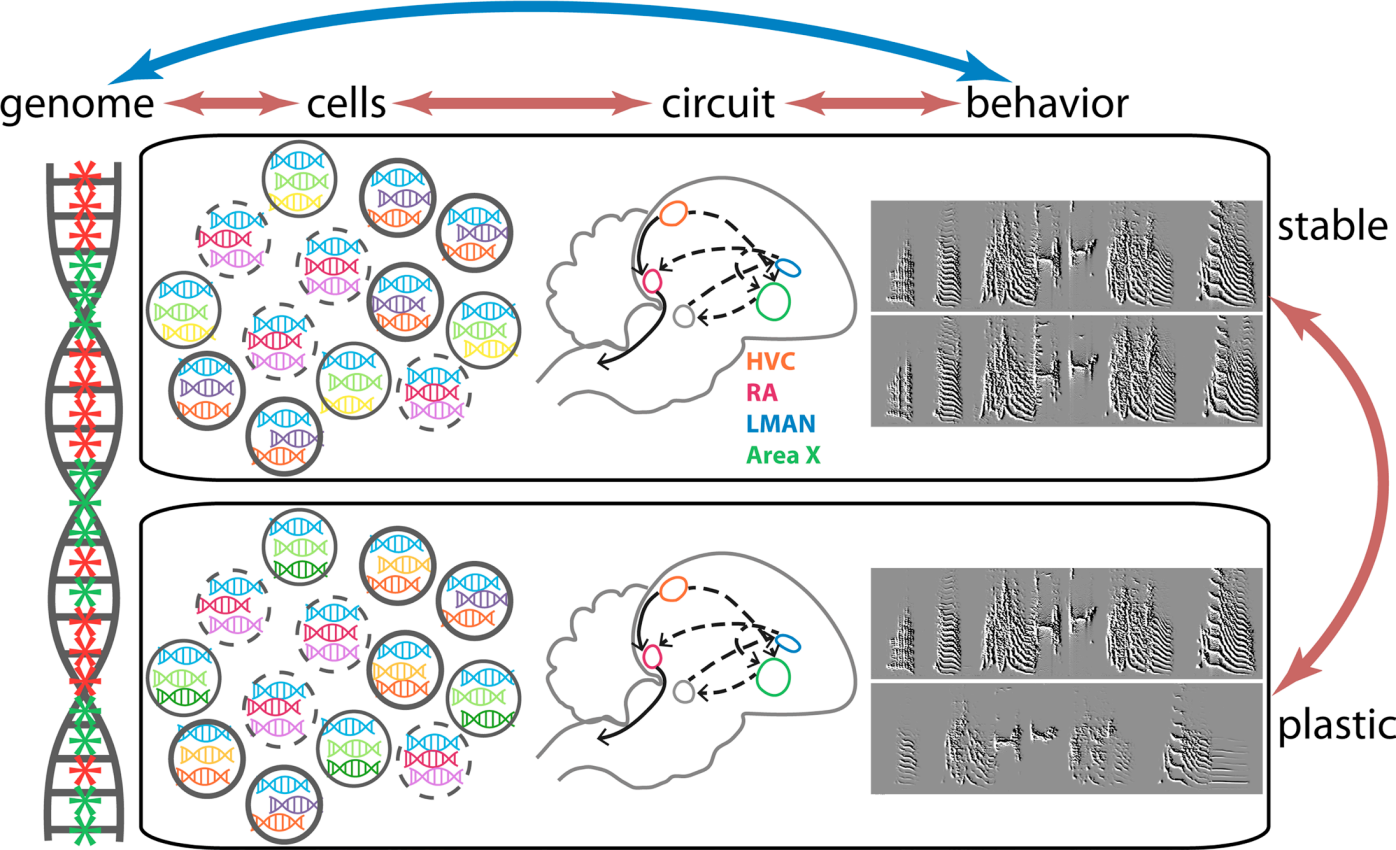
Changes in gene expression and birdsong in finches. It is known that changes in gene expression can affect the behavior of an individual, and that changes in behavior influence patterns of gene expression (double-headed blue arrow). Colquitt et al. studied these changes at the level of cells in the neural circuit involved in birdsong, which is a learned behavior (double-headed orange arrows). The four major regions of the brain involved in birdsong – HVC (orange); RA (red); LMAN (blue) and Area X (green) – are connected to form the circuit shown by the dashed and solid grey arrows. At any given time, the genome (left) contains regions where genes are expressed (green asterisks) and regions where genes are not expressed (red asterisks). All cells of a given cell type share major patterns of gene expression: this is shown schematically by all the cells with bold boundaries expressing blue, purple and orange genes, and likewise for the cells with solid and dashed boundaries. The song of an adult songbird is very consistent (see the top two sonograms on the right). However, the lack of auditory input caused by deafening leads to the song becoming unstable and variable (bottom two sonograms). Colquitt et al. found that in addition to its impact on birdsong, deafening leads to changes in the patterns of gene expression: for instance, the cells with bold boundaries can now have blue, purple and orange genes, as before, or blue, yellow and orange genes, and that changes in gene expression depend on which brain region of the circuit the cells are in.

Now, in eLife, Bradley Colquitt, Michael Brainard and colleagues at the University of California, San Francisco report new insights into how sensory loss impacts birdsong by measuring gene expression in several major brain regions necessary for song production in Bengalese finches (Colquitt et al., 2023). Similar to what happens in humans, the vocalizations of these birds change over the course of their development, changing from sounds that are unstructured and variable to songs that are highly structured and consistent by adulthood. Nevertheless, auditory input remains crucial, and deafening leads to variations in the songs of adult birds.

In their experiments, Colquitt et al. studied the expression of genes in birds that could hear and birds that were deafened: this involved measuring which genes are expressed in all cells within a brain region, and combining this with the results of prior studies to assess which of these genes were expressed together in individual cells. This enabled the team to identify the brain regions, genes and cell types that were most affected by auditory loss. This is important, because changes in gene expression do not always produce the same effect; for example, a change made to the signaling molecules in inhibitory cells will have different consequences if made to the signaling molecules in excitatory cells.

Colquitt et al. applied this approach to four brain regions necessary for learned birdsong production: HVC, RA, LMAN and Area X (Figure 1). This revealed that these song regions displayed more similar changes in gene expression and were more affected by hearing loss than adjacent non-song regions, suggesting that this neural circuit shares some common features. In hearing birds, the song regions directly connected to each other tended to share a greater number of expressed genes than those that were more distally connected. Deafening reduced this pattern and increased the variability of birdsongs.

Next, Colquitt et al. tested the idea that one brain area can affect gene expression in another by lesioning LMAN, without which the song variability typically observed in adult deafened birds does not occur. When the team lesioned LMAN in hearing birds and assayed gene expression profiles in the other three brain areas, the biggest changes occurred in one of its direct connections, RA (Figure 1). As expected, the patterns of RA gene expression were different than in the deafened birds. This suggests that direct connections between brain regions can alter patterns of gene expression. However, lesions did not lead to any significant changes in birdsong stability, which makes it difficult to interpret the behavioral relevance of these results.

A circuit-level analysis also revealed that RA and Area X were most affected by hearing loss, but why exactly is not fully clear. It is possible that the changes in gene expression in RA could be tied to behavioral alterations, as RA (together with HVC) is part of the motor pathway that birds need to perform their song. Area X, on the other hand, is predominantly active during song acquisition, but is not needed to produce and maintain songs once they have been learned (Sohrabji et al., 1990; Scharff and Nottebohm, 1991); thus, a different explanation is needed as changes in gene expression in this part of the brain would not be expected to change with the songs of adult birds. Another possibility is that because both RA and Area X receive inputs from two areas of the brain circuit (HVC and LMAN) rather than one, they are twice as likely to be ‘hit’ by a disruption in information flow following hearing loss (Nottebohm et al., 1976; Mooney and Konishi, 1991; Vates and Nottebohm, 1995). However, more research is needed to test these theories.

Finally, the experimental design was not able to distinguish between the following three potential relationships between gene expression and behavior. One option is that the loss of sensory input caused changes in gene expression, which then drove the variations in birdsong. It could also be possible that the modifications in the vocalizations itself led to changes in gene expression, which are only then indirectly associated with deafening. A third possibility is that a combination of these mechanisms is at play during different phases of behavioral changes, or in different brain areas and cell types. Defining these relationships will provide additional insight into the neural properties required to acquire and sustain learned behavior.
